# SkinSensPred as a Promising in Silico Tool for Integrated Testing Strategy on Skin Sensitization

**DOI:** 10.3390/ijerph191912856

**Published:** 2022-10-07

**Authors:** Shan-Shan Wang, Chia-Chi Wang, Chun-Wei Tung

**Affiliations:** 1 Ph.D. Program in Environmental and Occupational Medicine, College of Medicine, Kaohsiung Medical University, Kaohsiung 80708, Taiwan; 2 Institute of Biotechnology and Pharmaceutical Research, National Health Research Institutes, Miaoli County 35053, Taiwan; 3 Department and Graduate Institute of Veterinary Medicine, School of Veterinary Medicine, National Taiwan University, Taipei 10617, Taiwan

**Keywords:** adverse outcome pathway, skin sensitization, machine learning, SkinSensPred, 3R

## Abstract

Skin sensitization is an important regulatory endpoint associated with allergic contact dermatitis. Recently, several adverse outcome pathway (AOP)-based alternative methods were developed to replace animal testing for evaluating skin sensitizers. The AOP-based assays were further integrated as a two-out-of-three method with good predictivity. However, the acquisition of experimental data is resource-intensive. In contrast, an integrated testing strategy (ITS) capable of maximizing the usage of laboratory data from AOP-based and in silico methods was developed as defined approaches (DAs) to both hazard and potency assessment. There are currently two in silico models, namely Derek Nexus and OECD QSAR Toolbox, evaluated in the OECD Testing Guideline No. 497. Since more advanced machine learning algorithms have been proposed for skin sensitization prediction, it is therefore desirable to evaluate their performance under the ITS framework. This study evaluated the performance of a new ITS DA (ITS-SkinSensPred) adopting a transfer learning-based SkinSensPred model. Results showed that the ITS-SkinSensPred has similar or slightly better performance compared to the other ITS models. SkinSensPred-based ITS is expected to be a promising method for assessing skin sensitization.

## 1. Introduction

Skin sensitization is a key regulatory endpoint that evaluates allergic effects by chemical exposure [1]. The standard in vivo assays to evaluate skin sensitization are the guinea pig maximization test (GPMT, [2]) and murine local lymph node assay (LLNA, [3]). In 2013, the European Union’s ban on animal testing for cosmetic ingredients promoted the growth of non-animal testing methods. A few alternative methods have been validated and endorsed by the Organisation for Economic Co-operation and Development (OECD) including Test No. 442C [4], Test No. 442D [5], and Test No. 442E [6]. In addition, several in silico tools have been proposed for the prediction of skin sensitizers [7,8,9]. The tools can be classified as three categories: read-across, structural alerts, and machine learning-based methods. As each adverse outcome pathway (AOP)-based assay addresses only a specific key event (KE), a single assay alone may not be sufficient for replacing animal testing. It is therefore desirable to integrate multiple pieces of evidence from complementary assays to inform decision making.

Several attempts have been made to develop integrated testing strategies (ITS) combining multiple assay results to build a rule-based approach [10,11,12]. Recently, the defined approaches (DAs) for skin sensitization have been endorsed by OECD [13]. Three DAs were included in the testing guideline including two-out-of-three (2o3), ITSv1, and ITSv2. The 2o3 strategy makes a decision based on two concordant results from assays addressing KE1, KE2, and KE3 to facilitate hazard identification. While the 2o3 strategy is successful, the experiments required for reaching a final decision are resource-intensive. To maximize the usage of laboratory data, two scoring methods of ITSv1 and ITSv2 were proposed for both hazard identification and potency classification by considering assays from KE1, KE3, and in silico methods. Derek Nexus [14] and OECD QSAR Toolbox [15] were adopted in ITSv1 and ITSv2, respectively. Derek Nexus software is an expert rule-based system, while OECD QSAR Toolbox software provides workflows for skin sensitization [16]. In a comparison with ITS prediction and the Globally Harmonized System of Classification and Labeling of Chemicals (GHS) [17], both ITSv1 and ITSv2 showed promising results for classifying skin sensitizers. The DAs were also useful for classifying mixtures consisting of agrochemicals [18]. 

While the DAs performed well, only read-across and expert rule-based methods were evaluated. Recently, several advances in machine learning algorithms have been made by incorporating data from AOP-based assays that further improved the prediction of skin sensitization [9,19,20,21]. It is therefore interesting to evaluate AOP-based in silico methods as an alternative in silico tool under the DA framework. This study reported the performance by incorporating SkinSensPred as an alternative in silico tool of ITS. SkinSensPred [20] is an AOP-based transfer learning model for predicting human skin sensitization. For hazard and potency prediction, the proposed ITS-SkinSensPred showed similar or slightly better performance compared to the other ITS models. Furthermore, its application to mixtures of agrochemicals showed a 4% improvement in terms of balanced accuracy. ITS-SkinSensPred showed promising performance on datasets of single compounds and mixtures and is expected to be useful for analyzing skin sensitization of chemicals.

## 2. Materials and Methods

### 2.1. Dataset

For comparison, the dataset collected from the supplementary data of OECD guidelines of DA for skin sensitization [13] was applied to evaluate the proposed ITS-SkinSensPred. The dataset contained experimental data for 196 unique chemicals, including 66 human patch predictive test (HPPT) and 168 LLNA assay results as well as alternative assay results including the direct peptide reactivity assay (DPRA), KeratinoSens, and h-CLAT. Both binary hazard classification and potency subcategorization data were available for the evaluation of hazard and potency prediction. 

In addition, Strickland et al. extended the applicability of DA to 27 mixtures of agrochemicals [18], considering only active ingredients. In their study, the animal assay data without human evidence were compared with the adjusted DA approach, and individual methods include the DPRA assay, KeratinoSens assay, h-CLAT assay, and QSAR Toolbox. The commercial software Derek and the corresponding ITSv1 approach were not included in the comparison due to the tool being commercial. To predict the mixture, Strickland et al. [18] followed OECD guidelines [4,5,6] with minor modifications regarding conducting the in chemico and in vitro experiments. In addition, they followed the GHS guidance for in silico results, which classify a sensitizer mixture by at least one sensitizer ingredient whose concentration is more than 0.1%. We apply the same criterion for classifying mixtures. 

### 2.2. ITS-SkinSensPred

The ITS-defined approaches are score-based systems that weigh the evidence of two assays, DPRA and h-CLAT, and an additional in silico tool [13,18]. The proposed ITS-SkinSensPred utilizes the same criteria for calculating the scores of DPRA and h-CLAT assays [13,18] while replacing the in silico component as SkinSensPred. In brief, a score ranging from 0 to 3 was given according to DPRA and h-CLAT assay results of mean percent depletion for the cysteine and lysine peptides and minimum induction threshold, respectively. For in silico prediction, each test chemical was represented as a simplified molecular-input line-entry system (SMILES) string for in silico prediction of SkinSensPred [20]. SkinSensPred utilized a multitask extratree algorithm for improving the prediction of skin sensitization by simultaneously learning four tasks: protein binding, keratinocyte activation, activation of dendritic cells, and human skin sensitization. The training dataset was collected from SkinSensDB [22]. The webserver is available at https://cwtung.nhri.edu.tw/skinsensdb/predict (accessed on 1 September 2022). Given the SMILES string of a test chemical, the webserver returned the prediction score for skin sensitizer and applicability domain information. Only predictions within the defined applicability domain were considered in this study. A prediction score greater than 0.5 was considered a skin sensitizer. In contrast, a non-sensitizer prediction was made by SkinSensPred. The prediction of SkinSensPred was transformed to a binary score, where 1 point and 0 points were assigned for sensitizer and non-sensitizer, respectively. The summation of all scores was used to interpret the final hazard or potency classification. We adopted the same classification rules of ITS approaches [13,18] for comparison. For hazard identification, a chemical with a total score ranging from 2 to 7 was classified as a skin sensitizer. On the other hand, a total score less than or equal to 1 indicated a non-sensitizer. For potency classification, a total score ranging from 6 to 7 was classified as category 1A. A total score ranging from 2 to 5 was classified as category 1B. The rest were not classified (NC). Please note that missing inputs or prediction outside of the domain led to inconclusive prediction. 

### 2.3. Performance

The performance of hazard prediction is a summary of the numbers between prediction and experimental data as true positives (TP), true negatives (TN), false positives (FP), and false negatives (FN). The accuracy, sensitivity, specificity, and balanced accuracy were then calculated based on the numbers of TP, TN, FP, and FN. For potency prediction, accuracy was calculated for each subcategory and an overall accuracy was calculated for all chemicals. The performance measurements were computed using the following equations:

Accuracy = (TP + TN)/(TP + TN + FP + FN),(1)

Sensitivity = (TP)/(TP + FN),(2)

Specificity = (TN)/(TN + FP),(3)

Balanced accuracy = (Sensitivity + Specificity)/2.(4)

All analyses were implemented using the R programming language and packages dplyr and caret to process the data and calculate the performances [23,24].

## 3. Results and Discussion

### 3.1. Hazard Identification

An evaluation of ITS-SkinSensPred on the prediction of HPRT and LLNA skin sensitization was first conducted. The performance of ITS-SkinSensPred for hazard identification is summarized in Table 1. The reference datasets include 66 HPRT data and 168 LLNA data, where 83% (55/66) and 80% (135/168) of chemicals, respectively, were sensitizers. The balanced accuracy, sensitivity, and specificity of ITS-SkinSensPred on HPRT data are 70%, 94%, and 45%, respectively. ITS-SkinSensPred is better than ITSv1, with a 1% improvement on the three measurements of balanced accuracy, sensitivity, and specificity. In comparison to ITSv2, ITS-SkinSensPred provides a similar or slightly better performance. All ITS methods performed better than animal-based LLNA and worse than resource-consuming 2o3. As SkinSensPred was developed for predicting human skin sensitization, the results fit our expectation. As for the prediction of LLNA outcomes, ITS-SkinSensPred provides comparable results to ITSv1 and ITSv2 with a slightly increased specificity and a slightly decreased sensitivity. As the majority of the LLNA datapoints are sensitizers, the decrease in sensitivity resulted in a balanced accuracy of 80% that was slightly worse than ITSv1 and similar to ITSv2. All ITS methods are only slightly worse than 2o3 for predicting LLNA outcomes but require much less resources. All ITS approaches provide a coverage of greater than 90%, which is much higher than that of 2o3. Please refer to Table S1 for the detailed performance of hazard identification. 

Generally, the three in silico tools performed similarly under the ITS framework due to the design of the ITS scoring scheme, where only one point is contributed by in silico models while alternative assays contribute a maximum of three points. All three ITS methods have a good sensitivity of more than or equal to 88% for predicting HPRT data and LLNA data. As for specificity, all three models are much worse than 2o3, especially for human data. Please note that the data for evaluating performance of ITS methods are imbalanced, with only a few non-sensitizers. The evaluation of specificity for ITS methods may require further investigation by incorporating more non-sensitizers. Overall, ITS-SkinSensPred has a performance comparable to other ITS methods and is slightly better than other ITS methods for predicting human data.

### 3.2. Potency Prediction

For human data, the overall correct classification rates of ITSv1, ITSv2, and ITS-SkinSensPred are 68%, 70%, and 70%, respectively. A maximum 4% difference was observed in the comparison of the correct classification rate of groups 1A, 1B, and NC for the three ITS methods. Similar to that of hazard identification, ITS-SkinSensPred has the highest correct classification rate for each group, as shown in Table 2. All ITS models provide better performance than animal-based LLNA tests. The overall correct classification rate of ITS-SkinSensPred is 10% higher than LLNA. 

For LLNA data, an overall correct classification rate of 71% was obtained for all three ITS methods. ITSv1, ITSv2, and ITS-SkinSensPred provide best performances for group 1A, 1B, and NC, respectively. The highest correct classification rate for NC by ITS-SkinSensPred is consistent with the highest specificity for hazard identification (Table 1). Detailed information on model performance for potency classification is shown in Table S2.

### 3.3. Classification of Mixtures of Agrochemicals

The performance of ITS methods for predicting skin sensitization of mixtures of agrochemicals was evaluated for further comparison. The same rules were applied to evaluate a mixture with multiple substances, that is, a mixture is predicted as a sensitizer if any of the substances are predicted as skin sensitizers [18]. As shown in Table 3, the 2o3 method performed best with balanced accuracy, sensitivity, and specificity of 78%, 90%, and 67%, respectively. Among the evaluated ITS methods, ITS-SkinSensPred is better than ITSv2 in terms of balanced accuracy and specificity, where a 4% and 10% improvement, respectively, has been made. In contrast, the sensitivity of ITSv2 is 2% higher than that of ITS-SkinSensPred. Overall, ITS-SkinSensPred provides comparable sensitivity but much better specificity for the tested mixtures of agrochemicals, compared to ITSv2. As noted by the previous study [18], the h-CLAT assay may over-predict the tested agrochemicals containing endotoxins/liposaccharides, leading to a high sensitivity and low specificity. While a high coverage of 89% was obtained for ITSv2, its specificity and balanced accuracy were only 23% and 57%, respectively. The coverage of ITS-SkinSensPred is low compared to 2o3 and ITSv2. A previous study showed that pesticides with properties different from cosmetics and other industrial chemicals may not be readily predictable by current QSAR tools, whose training datasets contain only a few pesticides [25]. Further study addressing the lack of agrochemical data is desirable.

## 4. Conclusions

The DAs for skin sensitization are expected to reduce animal testing. To maximize the use of data from alternative assays, the scoring methods of ITS approaches based on two assays, DPRA and h-CLAT, and an in silico tool provide efficient and economic alternatives to the resource-consuming 2o3 method. In contrast to the commercial software of Derek Nexus and OECD QSAR Toolbox that require information technology expertise for installation and operation, the freely available online tool SkinSensPred provides an easily accessible option. Moreover, SkinSensPred is an AOP-based in silico model providing state-of-the-art performance for skin sensitization and is designed for predicting human skin sensitizers, which can provide complementary evidence to strengthen ITS prediction. This study evaluated the performance of the ITS-SkinSensPred approach for hazard and potency prediction. Our analysis results showed that ITS-SkinSensPred provides similar performance for hazard and potency prediction compared to that of ITSv1 and ITSv2. 

While good performance was achieved by the ITS-SkinSensPred approach, there are some limitations that should be considered. First, the in silico component of ITS approaches may not be applicable to inorganic compounds and metals that are commonly found in medical devices. Second, ITS approaches are generally more sensitive with potential false positives that should be taken into consideration when implementing ITS approaches for different regulatory purposes. Third, the application of ITS approaches and in silico methods for mixtures of agrochemicals may require more studies. Future works include the collection of more data for industrial chemicals for extending the coverage of SkinSensPred and development of algorithms for incorporating the effects from both active and other ingredients. Overall, the online and easily accessible tool SkinSensPred could be a promising in silico tool for ITS approaches.

## Figures and Tables

**Table 1:** Performance of hazard identification for LLNA data and human data.

	LLNA (*n* = 168)				Human (*n* = 66)			
Method	Balanced Accuracy (%)	Sensitivity (%)	Specificity (%)	Coverage (%)	Balanced Accuracy (%)	Sensitivity (%)	Specificity (%)	Coverage (%)
2o3 *	84	82	85	80	88	89	88	83
ITSv1 *	**81**	92	70	95	69	93	44	**97**
ITSv2 *	80	**93**	67	93	69	**94**	44	94
ITS-SkinSensPred	80	88	**72**	**96**	**70**	**94**	**45**	91
LLNA *	-	-	-	-	58	94	22	85

* Data were obtained from a previous study [13,18]. Bold numbers represent the best performance among the ITS approaches.

**Table 2:** Correct classification rate of potency classification for LLNA data and human data.

		LLNA (*n* = 156)				Human (*n* = 63)				
Method	Coverage (%)	Overall (%)	1A (%)	1B (%)	NC (%)	Coverage (%)	Overall (%)	1A (%)	1B (%)	NC (%)
ITSv1 *	**94**	**71**	**74**	71	70	**95**	68	65	77	44
ITSv2 *	90	**71**	72	**72**	67	90	**70**	**67**	80	44
ITS-SkinSensPred	**94**	**71**	73	69	**72**	89	**70**	**67**	**81**	**45**
LLNA *	-	-	-	-	-	75	60	56	74	25

* Data were obtained from a previous study [13,18]. Bold numbers represent the best performance among the ITS approaches.

**Table 3:** Performance of ITS methods for classifying mixtures consisting of agrochemicals.

Method	Balanced Accuracy (%)	Sensitivity (%)	Specificity (%)	Coverage (%)
2o3 *	78	90	67	70
ITSv2 *	57	**91**	23	**89**
ITS-SkinSensPred	**61**	89	**33**	67

* Data were obtained from a previous study [18]. Bold numbers represent the best performance among the ITS approaches.

## Data Availability

All data used in this study are available in the supplementary materials.

## Supplementary Materials

The following supporting information can be downloaded at: https://www.mdpi.com/article/10.3390/ijerph191912856/s1, Table S1: The detailed information on the performance of hazard identification for LLNA data and human data; Table S2: The detailed information on the confusion matrix and correct classification rate of potency for LLNA data and human data.; Table S3: Performance of DA approaches for classifying mixtures consisting of agrochemicals.
